# Surface roughness and Streptococcus mutans adhesion on surface sealant agent coupled interim crown materials after dynamic loading

**DOI:** 10.1186/s12903-022-02323-x

**Published:** 2022-07-19

**Authors:** Eda Eslemez Topcu, Onur Şahin, Ayşegül Köroğlu, Füsun Cömert, Burak Yilmaz

**Affiliations:** 1 Sincan Oral Dental Health Center, 06930 Ankara, Turkey; 2 Department of Prosthodontics, Faculty of Dentistry, Alanya Alaaddin Keykubat University, 07490 Antalya, Turkey; 3 Department of Prosthodontics, Faculty of Dentistry, Zonguldak Bülent Ecevit University, 67600 Zonguldak, Turkey; 4 Department of Microbiology, Faculty of Medicine, Zonguldak Bülent Ecevit University, 67600 Zonguldak, Turkey; 5 Department of Reconstructive Dentistry and Gerodontology, School of Dental Medicine, University of Bern, 3012 Bern, Switzerland; 6 Department of Restorative, Preventive and Pediatric Dentistry, School of Dental Medicine, University of Bern, 3012 Bern, Switzerland; 7 Division of Restorative and Prosthetic Dentistry, The Ohio State University, Columbus, OH 43210 USA

**Keywords:** Interim materials, Chewing simulator, Surface sealant agent, Surface roughness, Streptococcus mutans

## Abstract

**Background:**

With the application of surface sealant agents, smooth surfaces can be achieved in a shorter time when compared with conventional polishing. However, studies on the performance of these agents against chewing forces are not many. The purpose of this study was to evaluate the surface roughness and Streptococcus mutans adhesion on surface sealent coupled interim prosthetic materials after chewing simulation.

**Methods:**

One hundred and twelve specimens were fabricated from two poly(methyl methacrylate) (Tab 2000, Dentalon Plus) and two bis-acryl (Tempofit, Protemp 4) interim crown materials and divided into 4 groups (n = 7) according to applied surface treatment: conventional polishing (control) and 3 surface sealant (Palaseal, Optiglaze, Biscover) coupling methods. The surface roughness values (R_a_) were measured with a profilometer before (Ra0) and after aging through dynamic loading in a multifunctional chewing simulator for 10,000 cycles at 50 N load combined with integral thermocycling (between 5 and 55 °C) (Ra1). Specimens were incubated with Streptococcus mutans suspension and the total number of adherent bacteria was calculated by multiplying the counted bacterial colonies with the dilution coefficient.

**Results:**

Surface sealant agent application significantly decreased the surface roughness compared with conventionally polished specimens, except for Optiglaze or BisCover LV applied Protemp 4 and Palaseal or Biscover LV applied Tempofit. Surface roughness after dynamic loading showed a statistically significant increase in all groups, except for the control groups of Tab 2000 and Protemp 4. A positive correlation was found between surface roughness values of interim prosthodontic materials and the quantitiy of Streptococcus Mutans.

**Conclusions:**

Even though surface sealant agent application significantly decreased the surface roughness compared with conventionally polished specimens, dynamic loading significantly increased the surface roughness of all surface sealant coupled materials. The R_a_ values of all test groups were higher than the plaque accumulation threshold (0.20 µm). Streptococcus mutans adhered more on rougher surfaces.

## Background

Interim crowns provide function, aesthetics, protection of the pulp, prevent undesired tooth movements and gingival growth [1]. They may also be used to improve occlusal relationships in patients with non-ideal occlusion, when the vertical dimension is planned to be altered before the delivery of permanent restoration, and to the gingival shape, size, and location [2].

Polyethyl methacrylate (PEMA), poly (methyl methacrylate) (PMMA), urethane dimethacrylate (UDMA), polyvinyl methacrylate (PVMA), bis-acryl composite resin, and composite resin can be used with direct and indirect techniques to fabricate interim crowns [1–4]. Acrylic-based and composite-based materials, which have been used for many years, are still the most commonly used interim crown materials [2, 5].

Smooth surfaces are essential on restorations for aesthetics and reduced plaque accumulation [6, 7]. Interim crowns should be biocompatible and have surface properties that prevent bacterial adhesion and discoloration. Several studies have indicated that increased surface roughness promoted the increase in bacterial adherence and plaque accumulation [7–17].

In order to minimize the surface roughness on dental materials, polishing is carried out in several stages in laboratory conditions or chairside. Recently, surface sealant agents have been developed by manufacturers as an alternative to conventional polishing methods. These agents with different matrix formulations such as bisphenol glycidyl methacrylate (BisGMA), trimethylene glycol dimethacrylate (TEGDMA), tetrahydrofurfuryl methacrylate (THFMA) and urethane dimethacrylate (UDMA) have been developed to overcome surface defects, wear, and staining [18–21].

Restorative materials are expected to have good mechanical and surface properties, wear resistance, aesthetic appearance and biocompatibility. To understand the clinical performance of materials and their lifetime, dynamic loading can be applied by using chewing simulators. Wear after dynamic loading can impair the surface integrity, aesthetic appearance, positional stability and maxillomandibular relationship. In addition, due to wear, many systemic, biological and mechanical problems may occur through inhalation or ingestion of released monomer components or filler particles on material surface. In this context, the response of restorative materials to chewing simulation, certain loads and friction forces is crucial to investigate incidence of cracks and fractures, surface wear, and deterioration [22, 23]. Although it has been reported that surface sealant agents can provide smoother surfaces in a shorter time than conventional methods, studies on the condition of these agents after simulated chewing are lacking [18]. In addition, how different sealant agents interact with interim crown materials in varied compositions under loading is not known.

The present study aimed to evaluate the effect of different surface treatments on the surface roughness and *Streptococcus mutans* adherence on interim crown materials before and after dynamic loading in a multifunctional chewing simulator [24]. The first null hypothesis was that surface sealant agent coupling would not affect the surface roughness of interim crown materials. The second null hypothesis was that dynamic loading would not affect the surface roughness and *Streptococcus mutans* adherence on tested materials.

## Methods

### Specimen preparation and surface treatments

In the present study, two bis-acryl composite resin-based (Protempt 4 (Prt), Tempofit (Tmp)) and two auto-polymerized polymethyl methacrylate (Tab 2000 (Tab), Dentalon Plus (Dnt)) interim crown materials were evaluated. By using stainless steel molds, twenty-eight disc shaped (10 mm in diameter and 2 mm in thickness) specimens were prepared for each resin in accordance with the manufacturers’ instructions and divided into 4 groups (n = 7) for surface treatment procedures; conventional laboratory polishing (control) and application of 3 different surface sealant agents (Palaseal (Ps), Optiglaze (Og), Biscover LV (Bc)). Ssurface sealant agents and interim crown materials used in the present study are shown in Table 1.

**Table 1:** Surface sealant and interim crown materials used

Product	Code	Component	Manufacturer
Palaseal	Ps	Methyl methacrylate, tris(2-hydroxyethyl)-isocyanurate-triacrylate, acrylizedepoxyoligomer, acrylates, acrylizedpolysiloxane	Heraeus Kulzer GmbH
Optiglaze	Og	Methyl methacrylate, multifunctional acrylate, silica filler, photo inhibitor	GC Corp
Biscover LV	Bc	Dipentaerythritolpentaacrylate, ethanol	Bisco Inc
Tab 2000	Tab	Methyl methacrylate, n-butylmethacrylate	Kerr Corp
Dentalon Plus	Dnt	Methacrylate, copolymer, peroxide, initiator, pigment	Heraeus Kulzer GmbH
Protempt 4	Prt	Ethanol,2,2’-[(1-methylethylidene bis(4,1 phenyleneoxy)])] bis- diacetate,benzyl-phenyl- barbituric acid silane treated silica, tert-butyl	3 M ESPE
Tempofit	Tmp	Ethoxylated bisphenol A dimethacrylate	Detax

The surfaces of all specimens were finished with a tungsten carbide bur (S274 190 060, Horico) and wet-ground by a sanding machine (Phoenix Beta, Buehler) for 100 rev/min during 15 s, using 400 grit silicon carbide abrasive paper (Atlas Waterproof Sheet, Saint-Gobain). Control group specimens of each material were first polished using a slurry of coarse pumice (Isler Pomza, Isler Dental) and water with a bristle brush on a polishing lathe (P1000, Zubler) under standard pressure (for 90 s at a rate of 1500 rpm). Then, fine–polishing was achieved using a polishing paste (Universal Polishing Paste, Ivoclar Vivadent) and a lathe flannel wheel (Blaudent, Anka Dental) for 90 s. The specimens were coated with Palaseal, Optiglaze, or BisCover LV with a soft brush in a thin, even layer in one direction to avoid air bubble formation. Then, the specimens were polymerized (Dentacolor XS, Heraeus Kulzer GmbH) at a reading of 750 mW/cm^2^ for 90, 40, and 30 s, respectively.

### Chewing simulation and surface roughness assesments

Surface roughness assessments were performed by using a contact profilometer (Perthometer M2, Mahr). Three measurements were made on each specimen by moving the diamond stylus (NHT- 6) of the device 5 mm in 7 s across the specimen’s surface under constant pressure of 0.7 mN. The mean value of measurements obtained for each specimen was recorded as R_a_0 value in µm.

All specimens were subjected to an aging process consisting computerized dynamic loading test in a multifunctional chewing simulator (Mod Chewing Simulator, Esetron) for 10,000 cycles at 50 N load combined with integral thermocycling (between 5 and 55 °C). The descending and ascending velocities were 60 mm/s and the loading cycle frequency was 1.6 Hz. The antagonist tooth was simulated by stainless steel spherical ball, 6 mm in diameter. Following chewing simulation, surface roughness of specimens were remeasured. The measurements were repeated three times for each specimen and the means obtained for each specimen were recorded as R_a_1.

### Streptococcus mutans adhesion

Before bacterial adhesion, the specimens were cleaned with an ultrasonic cleaner (BioSonic; Coltène/Whaledent) for 15 min and sterilized in an autoclave at 121 °C for 15 min. Artificial saliva was prepared according to Fusayama formula: 0.4 g NaCl, 0.4 g KCl, 0.795 g CaCl_2_ (2H_2_O), 0.695 g Na_2_H_2_PO_4_ (H_2_O), 0.005 g Na_2_S (9H_2_O), 1 g CH_4_N_2_O [25]. Specimens were covered with artificial saliva and mucin suspension (M2378, Mucin from porcine stomach, Type II, Sigma Aldrich) (140 mg/100 ml) (5 ml) in a petri dish and left for 1 h to produce a pellicle [26, 27]. *Streptococcus mutans* NCTC 10,449 was used. and after rehydration of *Streptococcus mutans* strain in Tryptic Soy Broth (TSB, Oxoid), 100 µL of broth was transferred on to blood agar (Oxoid) and incubated in 5% CO_2_ ambient air at 37 °C for 24 h. Then, tubes containing 2 ml of *Streptococcus mutans* suspension with 0.5 of McFarland turbidity (10^8^ colony forming units/milliliter (CFU/ml)) were prepared in TSB (5% sucrose supplemented) and the incubated specimens in artificial saliva were transferred to those tubes. Tubes were incubated in same ambient conditions for 24 h. After incubation, the specimens were washed in sterile phosphate buffered saline (PBS) solution (8 gr NaCl, 0.2 g KCl, 1.44 g Na_2_HPO_4_ and 0.24 g KH_2_PO_4_ in 1000 ml of H_2_O) by centrifuge at 1000 g for 3 min. After centrifugation, each specimen was placed in new glass tubes containing 1 ml of sterile PBS. The bacterial adhesion was evaluated by measuring colony-forming units per mL (CFU/ml). The tubes were treated for 6 min in an ultrasonic bath (50 kHz and 150 W), thereby the adherent bacteria cells were allowed to pass into the PBS. Three 1/10 serial dilutions were made in order to obtain the lower quantity of bacteria in the sample. A 100 µl of diluted PBS samples was sealded on blood agar and incubated in 5% CO_2_ ambient at 37 °C for 24 h. At the end of incubation, the total number of adherent bacteria was calculated by multiplying the colonies observed counted with dilution coefficient.

### Scanning electron microscopy (SEM) analysis

The surfaces of all resin materials after dynamic loading were examined with a scanning electron microscope (SEM) (Nova Nano SEM 450, FEI Co.). The acceleration voltage of cathode was set to 15 kV at a working distance of 9–10 mm and imaging was performed with × 200, × 2000 and × 5000 magnifications. All images were examined by one observer.

### Statistical analysis

The data were statistically analyzed by using a software program (SPSS version 19.0; SPSS Inc.). Kolmorogov-Smirov test of homogeneity was used to evaluate the distribution of the variables. Surface roughness and bacterial adhesion data were analyzed with a 2-way ANOVA to evaluate the effects of surface treatment, resin type, and their interactions. The means were compared with Tukey HSD test (α = 0.05). Pearson correlation coefficient test was used to investigate the correlation between R_a_1 and bacterial adhesion values after dynamic loading, and *P* < 0.05 was considered significant.

## Results

According to the 2-way ANOVA, for R_a_, the effect of interim material and interim material-surface treatment interaction was statistically significant (*P* < 0.05) (Table 2). Mean R_a_0 and R_a_1 values and standard deviations for interim material-surface treatment combinations are shown in Table 3. When conventionally polished material groups were compared, significant differences were observed for R_a_0 values of Tab and Dnt groups, and for R_a_1 values of Dnt group (*P* < 0.05).

**Table 2:** Two-way ANOVA results for comparison of surface roughness (R_a_) and bacterial adhesion (CFU/ml)

Parameter	Source	SS	df	MS	F	*p*
R_a_	Interim material (A)	12.775	3	4.258	8.950	< 0.001*
	Surface treatment (B)	1.650	3	0.550	1.56	0.331
	A x B	14.919	9	1.658	3.484	0.001*
	Error	45.675	96	0.476		
	Total	468.137	112			
CFU/ml	Interim material (A)	60,067,676.670	3	20,022,558.890	152.179	< 0.001*
	Surface treatment (B)	2,594,065.955	3	864,688.652	6.572	<0 .001*
	A x B	7,002,976.509	9	778,108.501	5.914	< 0.001*
	Error	12,630,959.143	96	131,572.491		
	Total	217,765,083.000	112			

*SS* sum of squares; *df* degrees of freedom; *MS* mean square

*P < 0.05 indicates statistical significance

**Table 3:** Mean R_a_0 and R_a_1 values (μm), standard deviations (SD) and the statistical summaries of test groups

Interim Material	Surface Treatment	R_a_0		R_a_1		t-test** (*P* values)
		Mean (SD)	Tamhane*	Mean (SD)	Tamhane*	
Tab	Con	1.66 (0.39)	Cb	1.83 (0.37)	Aa	0.433
	Ps	0.31 (0.26)	Aa	1.35 (0.58)	Aa	0.002
	Og	0.52 (0.08)	ABa	2.29 (1.00)	Aa	0.004
	Bc	0.53 (0.15)	Aa	1.60 (0.44)	Aa	0.001
Dnt	Con	1.08 (0.26)	Bb	3.18 (0.64)	Bb	0.001
	Ps	0.32 (0.11)	Aa	1.91 (0.86)	Aa	0.003
	Og	0.40 (0.14)	ABa	2.50 (0.53)	Aab	0.001
	Bc	0.56 (0.18)	Aa	1.95 (0.46)	Aa	0.001
Prt	Con	0.74 (0.18)	Ab	0.91 (0.13)	Aa	0.066
	Ps	0.22 (0.09)	Aa	1.41 (0.64)	Aab	0.003
	Og	0.68 (0.24)	Bb	1.34 (0.71)	Aab	0.049
	Bc	0.47 (0.12)	Aab	2.12 (0.99)	Ab	0.004
Tmp	Con	0.81 (0.14)	Ab	1.88 (1.01)	Aa	0.030
	Ps	0.51 (0.18)	Aab	2.09 (0.54)	Aa	0.001
	Og	0.22 (0.11)	Aa	1.91 (0.53)	Aa	0.001
	Bc	0.48 (0.19)	Aab	1.70 (0.88)	Aa	0.010

*Statistical comparisons between interim material/surface treatment groups were shown as letters and values having same letters are not significantly different for Tamhane test (*p* > 0.05). The capital letters indicates the comparisons between same surface treatment applied interim material groups and the small caps indicates the differences between surface treatment groups for the same interim material

**The pairwise comparisons of R_a_0 and R_a_1 values with independent sample t-test (*P* < 0.05 indicates statistical significance)

**Fig. 1:**
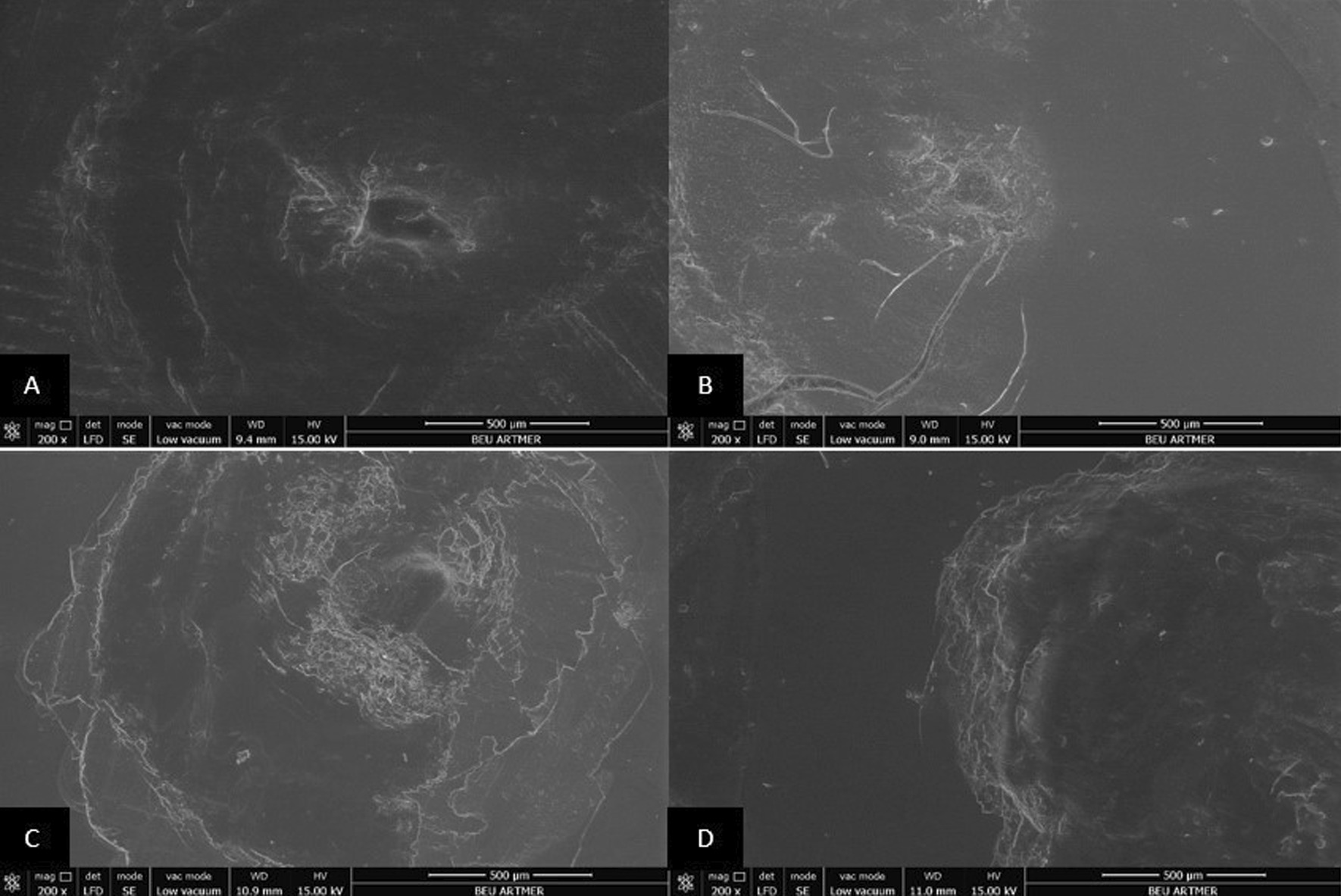
Scanning electron micrograph analysis after dynamic loading process (× 200 magnification). Conventionally polished (**A**), Palaseal (**B**), Optiglaze (**C**), BisCover LV (**D**) coupled Tab 2000 interim crown materials

**Fig. 2:**
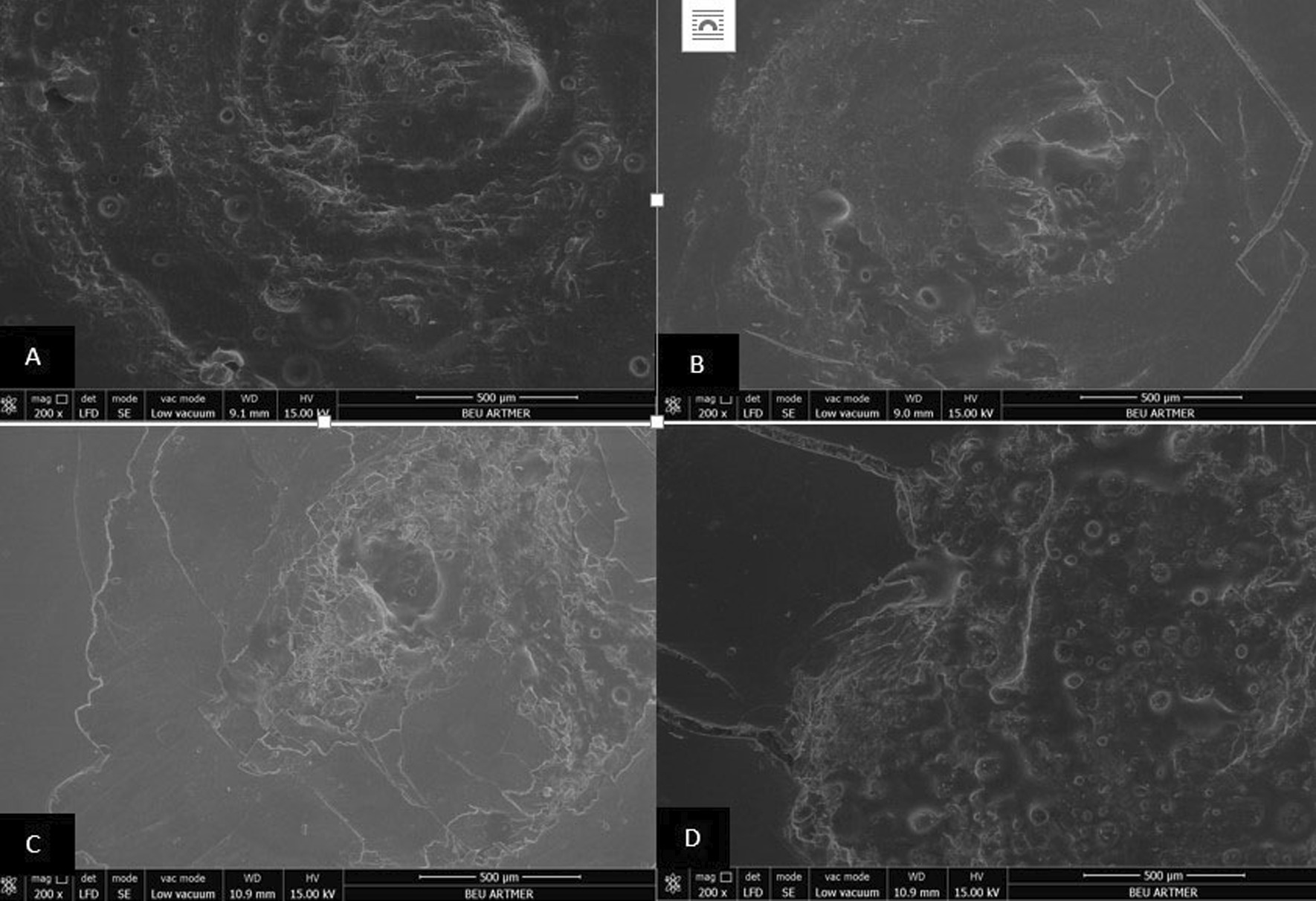
Scanning electron micrograph analysis after dynamic loading process (× 200 magnification). Conventionally polished (**A**), Palaseal (**B**), Optiglaze (**C**), BisCover LV (**D**) coupled Dentalon Plus interim crown materials (note rougher surface for conventionally polished specimen)

**Fig. 3:**
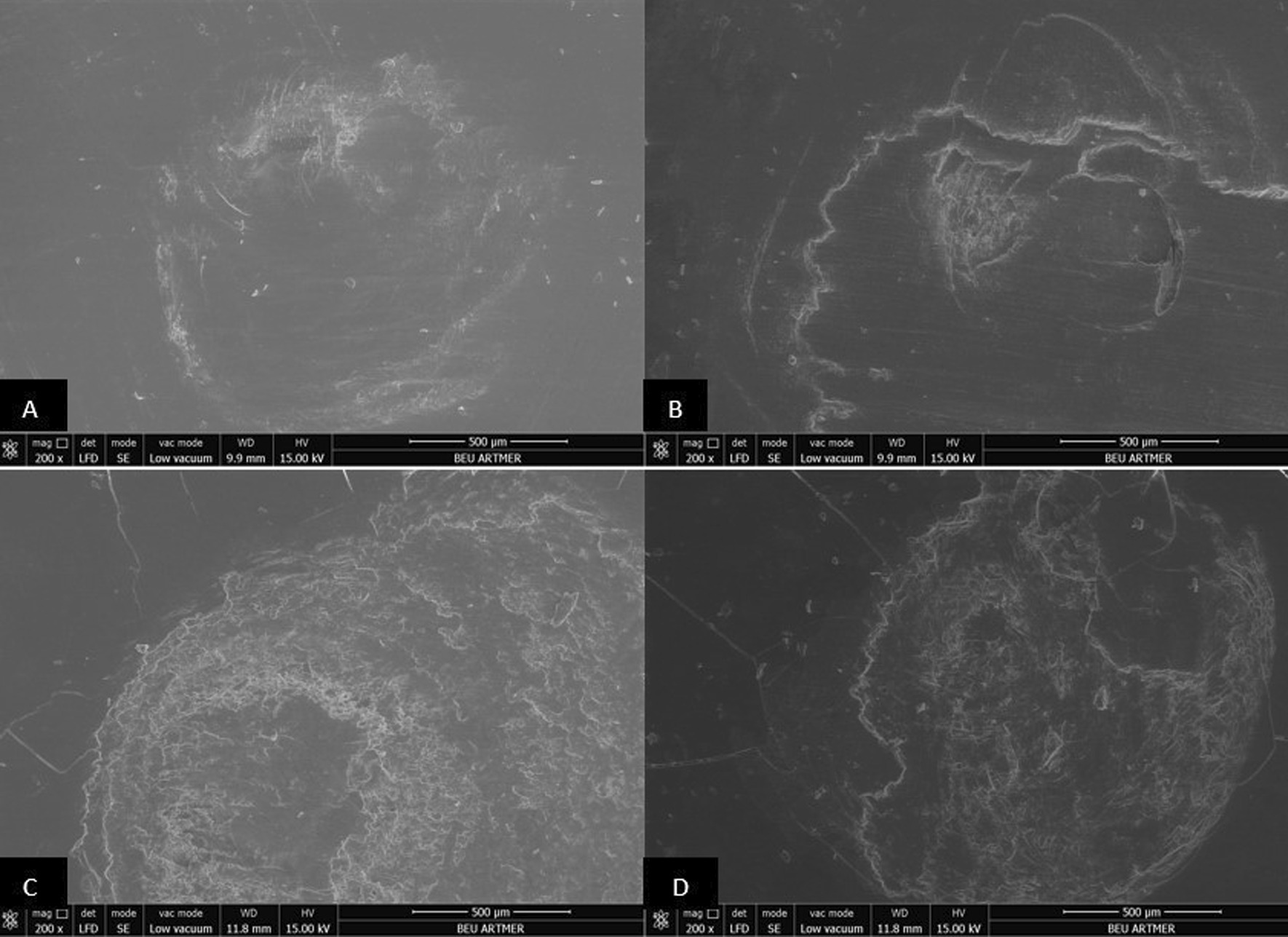
Scanning electron micrograph analysis after dynamic loading process (× 200 magnification). Conventionally polished (**A**), Palaseal (**B**), Optiglaze (**C**), BisCover LV (**D**) coupled Protemp 4 interim crown materials (note rougher surface for Biscover LV coupled specimen)

**Fig. 4:**
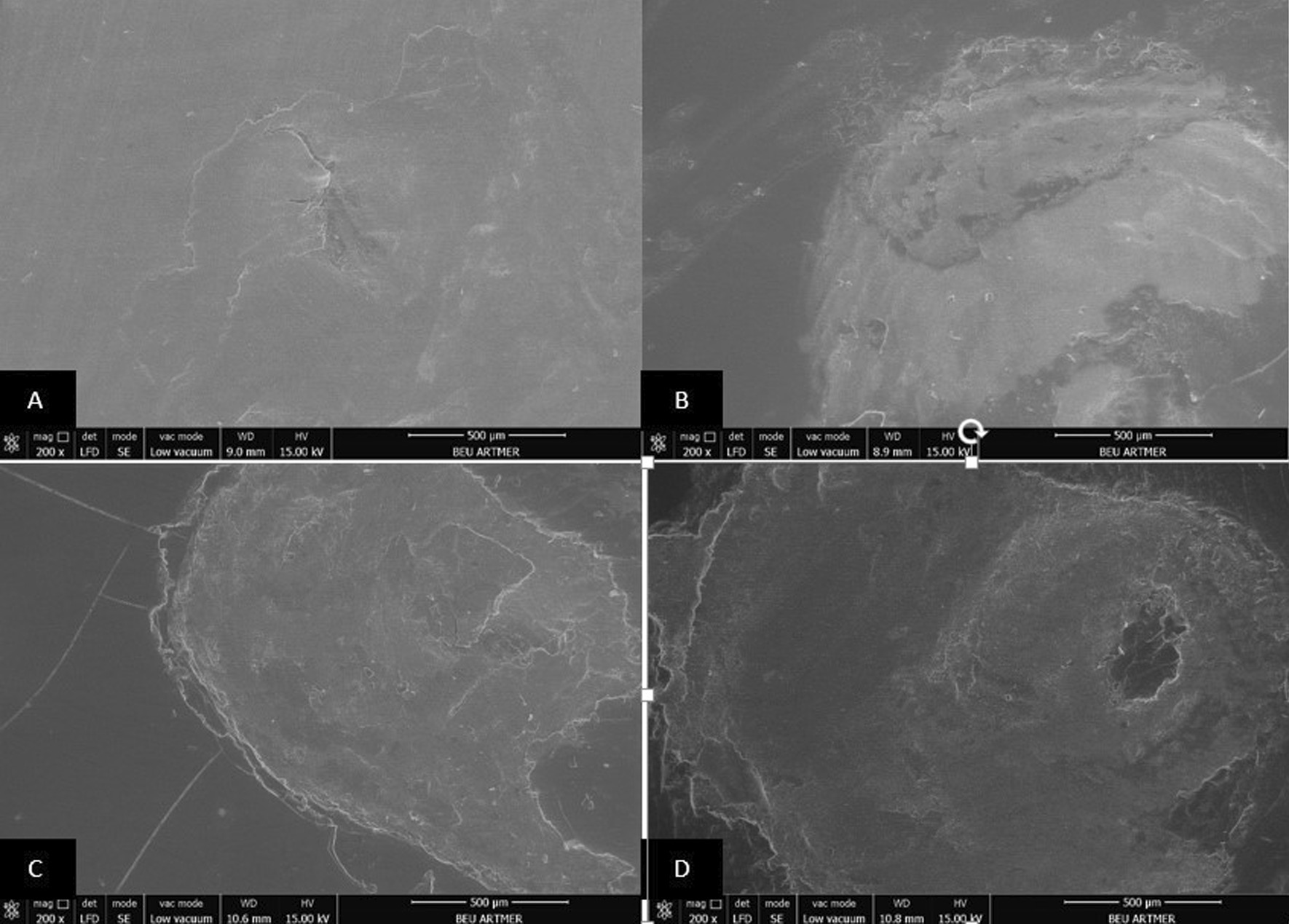
Scanning electron micrograph analysis after dynamic loading process (× 200 magnification). Conventionally polished (**A**), Palaseal (**B**), Optiglaze (**C**), BisCover LV (**D**) coupled Tempofit interim crown materials

For R_a_0 values, statistically significant differences were observed between the control and surface sealant applied specimens of all materials, except for Prt_Og, Prt_Bc, Tmp_Ps and Tmp_Bc (*P* < 0.05). Following dynamic loading, for R_a_1 values, statistically significant differences were found between Dnt control and Ps_Dnt and Bc_Dnt, and between Prt control and Prt_Bc (*P* < 0.05). Except for control groups of Tab and Prt, differences between R_a_0 and R_a_1 values were statistically significant in all groups (*P* < 0.05). The R_a_0 values (0.22 to 1.66 µm) and the R_a_1 values (1.34 to 3.18 µm) for all groups were higher than the plaque accumulation threshold (0.20 µm). SEM images of the surfaces of Tab, Dnt, Prt, and Tmp after dynamic loading are shown in Figs. 1, 2, 3 and 4.

**Fig. 5:**
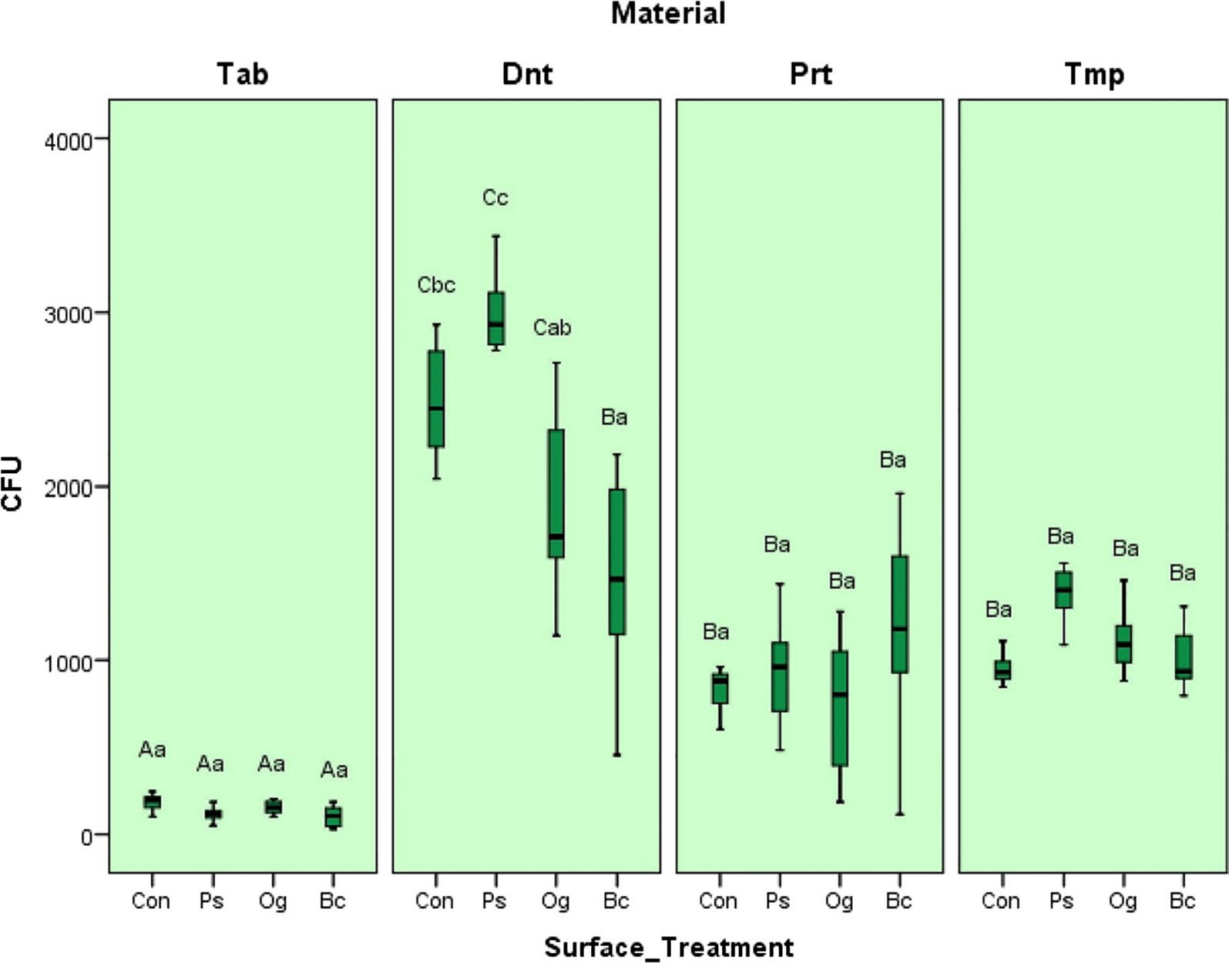
Mean CFU values (CFU/ml) of test groups

The interim material, surface treatment, and their interaction were statistically significant for bacterial adhesion (*P* < 0.001). Mean CFU/ml values, standard deviations (SD) and the statistical summaries for the interim material-surface treatment technique combinations are shown in Table 4. For all interim material groups, no statistically significant differences were observed between the control group and the surface sealant agent-coupled groups, except for Dnt_Bc (*P* > 0.05) (Fig. 5).

**Table 4:** CFU/ml values and statistical summaries of test groups

Interim material	Surface treatment	CFU/ml	
		Mean (SD)	Tamhane*
Tab	Con	177.14 (55.37)	Aa
	Ps	111.43 (41.81)	Aa
	Og	150.00 (41.53)	Aa
	Bc	99.29 (60.72)	Aa
Dnt	Con	2487.86 (348.59)	Cbc
	Ps	2887.14 (459.19)	Cc
	Og	1912.29 (563.62)	Cab
	Bc	1480.57 (621.02)	Ba
Prt	Con	980.00 (486.21)	Ba
	Ps	927.14 (340.13)	Ba
	Og	734.86 (430.32	Ba
	Bc	1186.86 (638.34)	Ba
Tmp	Con	950.00 (92.60)	Ba
	Ps	1382.14 (172.60)	Ba
	Og	1114.29 (199.26)	Ba
	Bc	1015.71 (190.84)	Ba

*Statistical comparisons between interim material/surface treatment groups were shown as letters and values having same letters are not significantly different for Tamhane test (*P* > 0.05). The capital letters indicate the comparisons between same surface treatment applied interim material groups and the small letters indicate the differences between surface treatment groups for the same interim material

**Fig. 6:**
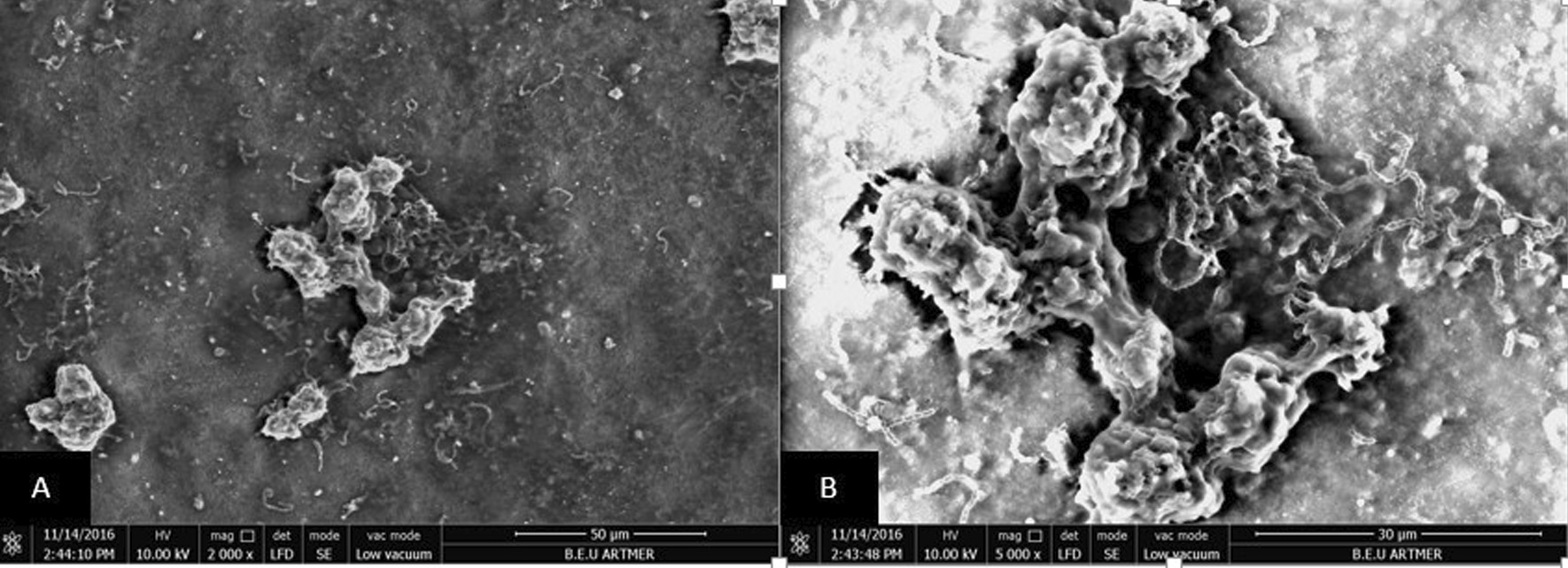
Scanning electron micrograph analysis after Streptococcus Mutans adhesion and proliferation (by two different magnifications, note aggregation on a rough surface). **A** 2000 × magnification; **B** 5000 × magnification

According to the Pearson Correlation Analysis, the coefficient of correlation between R_a_1 and Streptococcus mutans adhesion was statistically significant (*P* < 0.001, r^2^ = 0.323) and indicated that these two variables were moderately correlated (Table 5). SEM images of *Streptococcus mutans* adhesion and proliferation on rough surfaces are shown in Fig. 6.

**Table 5:** Correlation between R_a_1 and bacterial colonisation

		R_a_1	Colonisation
R_a_1	Pearson correlation	1	0.323**
	Sig.		0.001
	N	112	112
Colonisation	Pearson correlation	0.323**	1
	Sig.	0.001	
	N	112	112

**Correlation is significant for *P*˂ 0.01 level

## Discussion

The first null hypothesis of this study was rejected because the effect of surface sealant agent coupling was significant on the surface roughness of some interim crown materials. Dynamic loading was significant on the surface roughness of interim crown materials except for control groups of Tab and Prt. Also, *Streptococcus mutans* adherence on interim crown materials was affected by dynamic loading. Accordingly, the second null hypothesis was rejected.

Rough surfaces lead to staining of the restoration, dental plaque formation, and the adhesion of oral microorganisms promotes tooth loss due to carious lesions and periodontal disease [8–15]. The increase in surface roughness promotes bacterial adhesion by increasing the contact area between the surface and microbial cells. Restorations with rough surfaces increase plaque accumulation by promoting the retention, survival and proliferation of Streptococcus mutans, which are considered to be one of the main pathogens involved in bacterial colonization and development of secondary caries, and many other microorganisms [28–30].

In the present study, R_a_0 values of interim crown materials ranged between 0.22 and 1.66 µm, which were below the clinical undetectability limit of 10 µm that Kaplan et al. [31] reported. However, these values are above the threshold R_a_ of 0.20 µm that Bollen et al. [32] indicated. Similar to the study findings of Ayuso-Montero et al. [8], the control groups of PMMA resins showed higher surface roughness values compared with the control groups of bis-acryl composite resins. Contrarily, unlike the findings of the present study, Şen et al. [3] reported that due to the heterogeneous composition of bis-acrylate composite resins, higher surface roughness values were observed with filler particles extruding on the surface.

Surface sealant agents applied to the surfaces of materials in a single phase are more advantageous than conventional polishing processes in terms of application and time. Surface sealant agents contribute to surface smoothness by filling the surface defects and micro cracks after application. However, due to their high viscosity, there are disadvantages such as weak bonding to the underlying material, degradation of surface quality, and low resistance to abrasion [18, 33]. In the present study, when the R_a_0 values were evaluated, similar to previous studies [18, 20], surface sealant agent application decreased the Ra values of all PMMA resin groups and bis-acryl resin groups, except Og- or Bc-coupled Prt and Pc- or Bc-coupled Tmp groups. Statistically significant differences observed in PMMA resins may have been due to increasing effect on the molecular weight of the components present on the surface of the methacrylate, and the decrease in surface roughness with the application of surface sealant agent. However, it has been shown that surface sealant agent application could remove surface particles that are not polymerized or adhered to the surface, causing surface irregularities. Also, application errors and the formation of air bubbles were reported to increase the R_a_ [19].

Due to intraoral conditions including chewing forces, nonpolymerized layer may be separated from the surface and micro-cracks may occur [21]. In the present study, dynamic loading was performed with multidirectional chewing simulator in order to evaluate the effect of oral environment on specimens prepared from interim crown materials. During dynamic loading, 6 mm diameter steatite, which has similar physical properties to enamel, was used as an antagonist [34]. It is recommended that 240,000 chewing cycles should be performed to simulate 1 year of clinical service [22]. In the present study, 10,000 cycles corresponding to 15 days of clinical use were applied and after the dynamic loading process, it was observed that the surface roughness increased in all surface sealant agent coupled specimens. Also, SEM images of all resin groups after dynamic loading were consistent with the surface roughness measurements. This result may be attributed to the surface defects that occurred due to easy removal of the layer, potentially incompletely polymerized, on the surface of the resin, and to the low resistance of sealant agents to abrasion [21].

*Streptococcus mutans* has been reported to be the most abundant bacteria on enamel and root plaque (77%) [14], has high adhesion to all surfaces in the mouth, and it is a bacteria that is virulent with its acidogenic and aciduric properties [11]. Although in vitro studies have shown that artificial saliva does not reflect all the features of natural saliva [33], its use is essential for standardization [28, 35]. In the present study, to enable bacterial adhesion on the surface of the specimens, artificial saliva was prepared in accordance with the equation of Fusayama [33].

Similar to the findings of the present study, Aykent et al. [9] reported a positive correlation between the surface roughness and bacterial adhesion of restorative materials polished with different procedures. In the present study, SEM images (Fig. 6) revealed bacterial aggregation in areas with high surface roughness. Haralur et al. [36] compared stainless steel crowns, PMMA and bis-acryl resin interim crown materials, and the highest dental plaque accumulation was observed on PMMA specimens and the least was observed on the stainless-steel crown. Bacterial adhesion and proliferation on PMMA and bis acryl resin groups were reported to be due to hydrophilic polymer matrix and monomer structure. In the present study, the highest bacterial adhesion was found in the Dnt_Ps specimen group, while the least bacterial adhesion was in the Tab_Bc group.

Although surface roughness is an important feature in terms of bacterial adhesion, it is not a sufficient factor alone [6]. The effects of physical properties of materials such as surface electrical properties and free energy, hydrophobicity, fluoride release, as well as chemical properties have been previously studied [15]. Quirynen et al. [16] reported more dental plaque deposition on hydrophilic surfaces than on hydrophobic surfaces. Olsson et al. [15] stated that there was a critical limit on the hydrophobicity of surfaces in dental plaque deposition and the deposition below this limit would be minimal. Pellicle coating of the surfaces of dental materials changes the surface energy, which changes the bacteriostatic or bactericidal effect of the dental plaque [17]. Accordingly, these factors should be taken into consideration when interpreting the effects of varying factors and situations.

The specimens used in the current study were prepared in disc form containing flat surfaces, however, the recesses and protrusions on the tooth morphology may not allow an effective polishing process, and the roughness and bacterial adhesion may be affected. Clinical studies are needed to corroborate the findings of the present study. Also, further in vitro and in vivo research is needed to evaluate other factors affecting bacterial adhesion to interim materials particularly when surface sealants are used.

## Conclusions

Within the limitations of this study, the following conclusions were drawn:

Even though surface sealant agent application significantly decreased the surface roughness compared with conventionally polished specimens, dynamic loading significantly increased the surface roughness of all surface sealant coupled materials. Ra_a_ values of all test groups were higher than plaque accumulation threshold (0.20 µm). Streptococcus mutans adhered more on rougher surfaces. Although tested surface sealant agents enabled smoother surfaces, their use on occlusal surfaces of tested interim crown materials may lead to increased roughness compared with conventional polishing. When Dentalon is used, Biscover LV application can be recommended for smoother surfaces with less bacterial adhesion.

## Data Availability

The datasets used and/or analysed during the current study are available from the corresponding author on reasonable request.

## Abbreviations

Bc: Biscover LV
BisGMA: Bisphenol glycidyl methacrylate
°C: Celsius
CFU/ml: Colony-forming unit/milliliter
Dnt: Dentalon Plus
mm: Millimeter
mN: MilliNewton
mm/s: Millimeter/seconds
ml: Milliliter
µl: Microliter
mW/cm^2^: Miliwats/centimeter squared
N: Newton
Og: Optiglaze
PBS: Phosphate buffered saline
PEMA: Polyethyl methacrylate
PMMA: Poly (methyl methacrylate)
Prt: Protempt 4
Ps: Palaseal
PVMA: Polyvinyl methacrylate
rpm: Revolutions per minute
Tab: Tab 2000
TEGDMA: Trimethylene glycol dimethacrylate
THFMA: Tetrahydrofurfuryl methacrylate
Tmp: Tempofit
SEM: Scanning electron microscopy
UDMA: Urethane dimethacrylate
